# Spontaneous Incomplete transverse subtrochanteric femoral fracture with cortical thickening possibly secondary to risedronate use: a case report

**DOI:** 10.1186/1752-1947-6-272

**Published:** 2012-09-03

**Authors:** Anas Alfahad, Ei Mon Thet, Fawzy Radwan, Joe Sudhakar, Khin Nini, Phaedra Tachtatzis

**Affiliations:** 1 Darlington Memorial Hospital, Hollyhurst Road, Darlington, DL3 6HX, United Kingdom

## Abstract

**Introduction:**

Osteoporosis is an asymptomatic disease characterized by bone weakening and predisposition to fragility (insufficiency) fractures and can have devastating effects on individual life and great financial impact on the economy. Bisphosphonates are used worldwide for the primary and secondary prevention of osteoporotic fractures. However, increasing evidence raises concern that bisphosphonates can be associated with atypical fractures.

**Case presentation:**

A 65-year-old Caucasian woman on long-term steroid treatment for polymyalgia rheumatica was admitted with severe and constant pain in the right hip, radiating to the right knee. She had a history of steroid-induced osteoporosis, for which she was started on risedronate four years earlier. She had no history of trauma. Her blood results were unremarkable. Her X-rays confirmed that she had an incomplete right subtrochanteric femoral fracture. A bone scan confirmed the diagnosis and also ruled out any other associated fractures. Our patient successfully underwent internal nail fixation of the fracture. She was reviewed by a rheumatology team, which stopped the risedronate. She was started on treatment with denosumab injection.

**Conclusions:**

Previous case series have reported that long-term bisphosphonate use is associated with atypical fractures of the femur, and certain criteria have been established to help identify such rare fractures. Delayed union or non-union is expected in such fractures following definitive orthopedic treatment because of the long half life of bisphosphonates. In this case report, we try to raise questions related to this important subject, like the duration and safety of bisphosphonate use and the alternative medications used in osteoporosis in this rare condition. We consider this case report not only interesting but also important and unusual because it is about a patient who developed a potentially rare and serious side effect of long-term bisphosphonate use, estimated to affect 2.3 in every 10,000 patients, and who presented with a pelvic X-ray that showed the characteristic features of atypical fractures secondary to risedronate use. In addition, most of the documented cases have been associated with many years of bisphosphonate use whereas our patient had been on risedronate for only four years.

## Introduction

People with osteoporosis are at risk of fragility (insufficiency) fractures. These are fractures that occur as a result of mechanical forces that ordinarily would not cause a fracture. The World Health Organization has quantified these forces as being equivalent to a fall from a standing height. Fragility fractures are frequently associated with substantial disability, pain, and reduced quality of life. In the absence of a fracture, osteoporosis is asymptomatic and often remains undiagnosed. In women older than 50 years of age, the lifetime risks are estimated to be about one in three for a vertebral fracture and one in six for a hip fracture.

Bisphosphonates are considered the first-line treatment for primary and secondary prevention of osteoporosis-associated fractures [1,2]. However, there has been concern regarding the safety of bisphosphonates following the emergence of several case series that attribute the long-term use of bisphosphonates to a certain type of atypical fracture.

We report a rare case of a possible complication of bisphosphonate use and take the opportunity to discuss the characteristics of bisphosphonate-induced fractures, the duration or time scale that should be implemented for its use, and the management of a patient presenting with such a fracture.

## Case presentation

A 65-year-old Caucasian woman, who was a retired social worker, was admitted with severe and constant right hip pain that radiated to the right knee for six weeks before admission. She had a background of polymyalgia rheumatica on long-term steroid treatment (started five years earlier) and steroid-induced osteoporosis and vertebral fracture, for which she had been taking risendronate for four years. She had no history of trauma or any obvious predisposing factors.

She has a medical history of hypothyroidism, hypertension, and osteoarthritis of the right knee. She is a non-smoker, does not drink alcohol, and was not on any other medications. She had no allergies of note.

**Figure 1:**
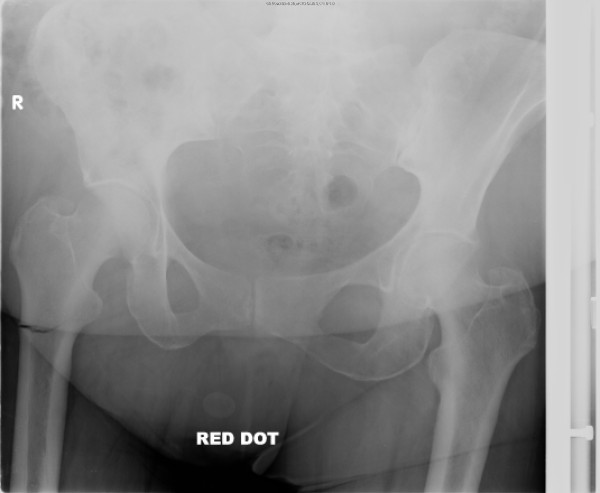
X-ray of the right hip showing an incomplete subtrochanteric fracture of right hip.

**Figure 2:**
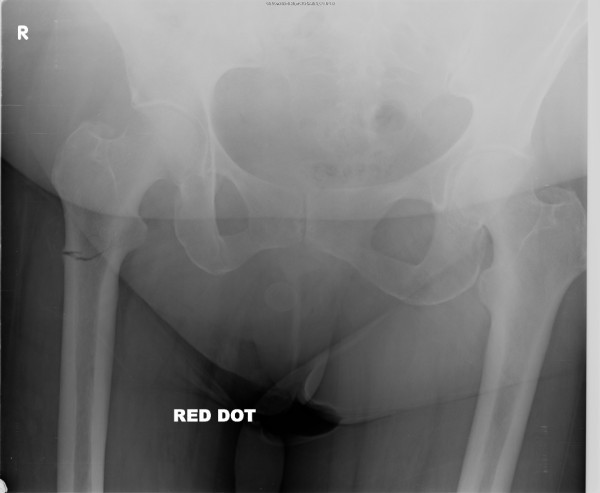
X-ray of the right hip showing an incomplete subtrochanteric fracture of right hip.

**Figure 3:**
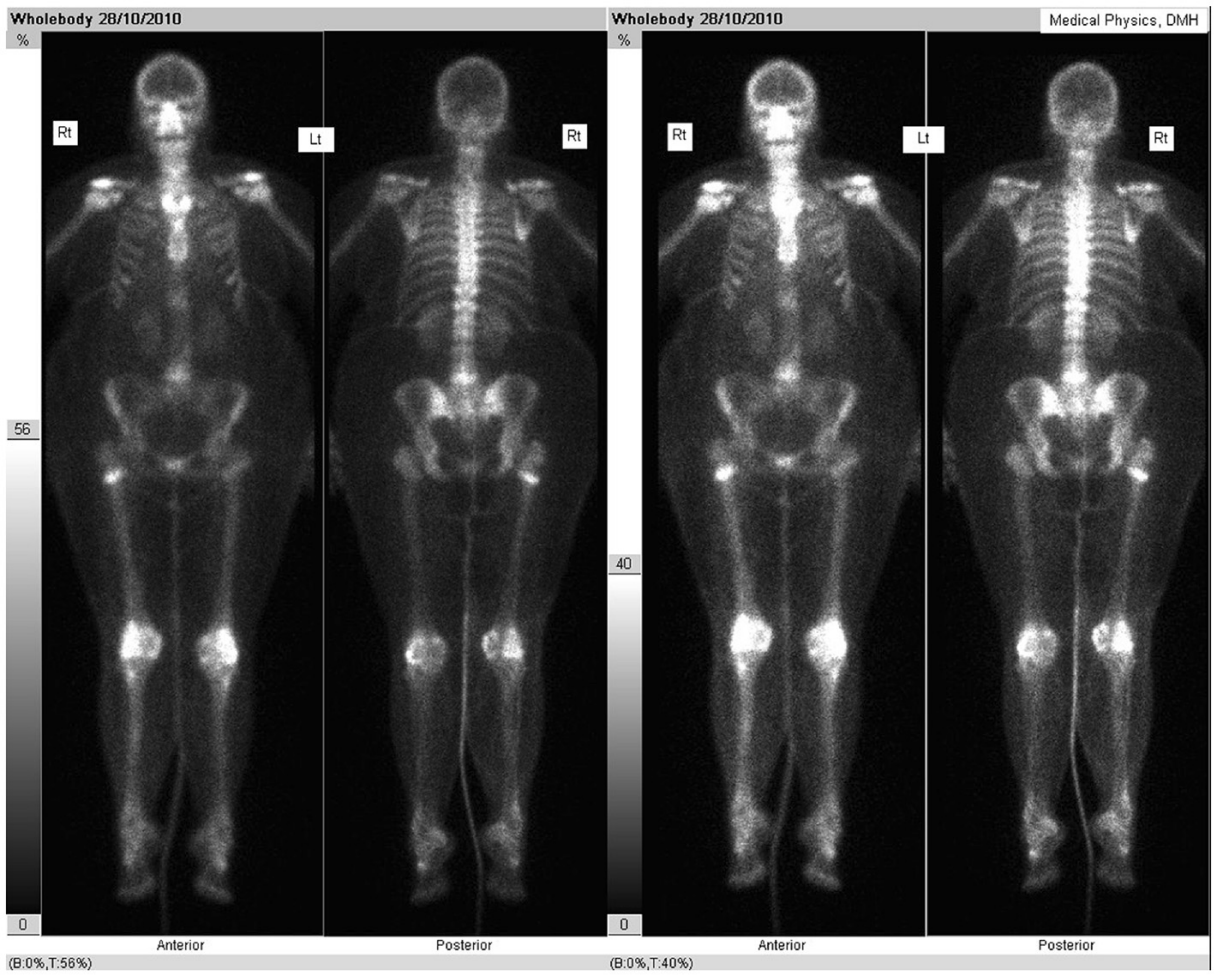
Bone scan showing increase uptake in the right hip.

During an examination, she had tenderness in the right trochanteric area and right hip. X-rays of the pelvis (Figures 1 and 2) showed an incomplete transverse subtrochanteric fracture of the right hip with bilateral cortical thickening. A bone scan confirmed the right hip fracture. A previous vertebral fracture and the arthritic activity in the shoulders and the knees were noted (Figure 3).

Her full blood count, electrolytes, coagulation, and bone profiles were normal.

**Figure 4:**
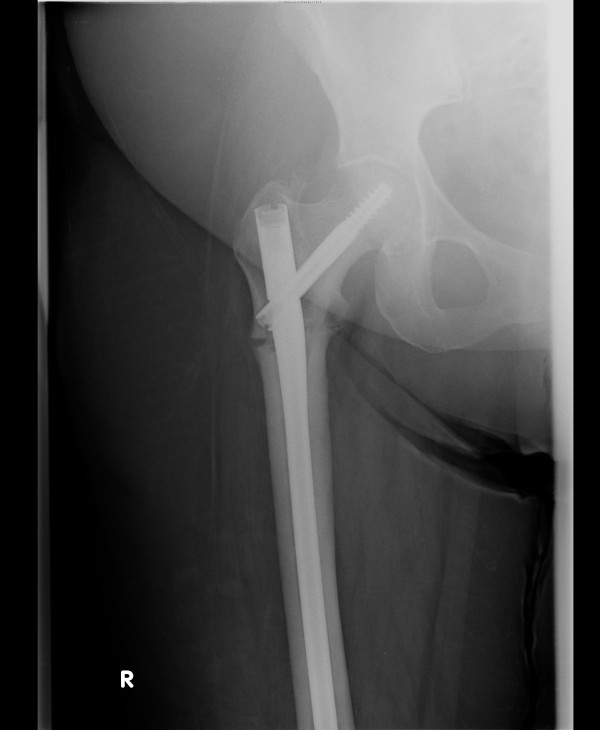
X-ray of the right hip immediately following long Gamma nail insertion.

**Figure 5:**
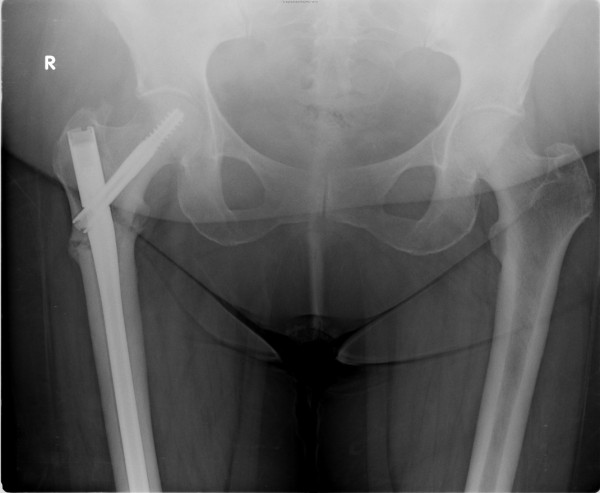
X-ray of the right hip six months following Gamma nail insertion with delayed bone healing process.

**Figure 6:**
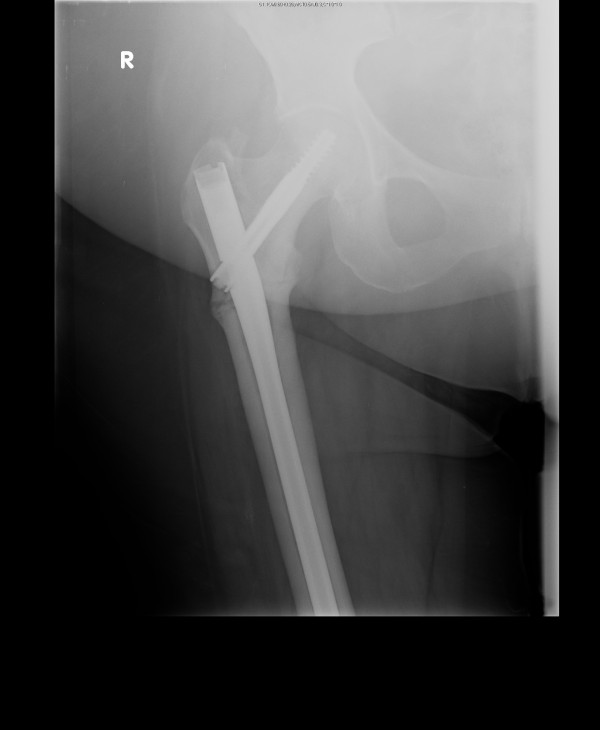
X-ray of the right hip 12 months following Gamma nail insertion with delayed bone healing process.

The fracture was fixed with a long Gamma 3 nail that was inserted into her right femur, and she was referred for physiotherapy (Figure 4). She was seen by the rheumatology team, which diagnosed a bisphosphonate-precipitated femoral fracture; risedronate was discontinued and she was started on denosumab instead. She was followed up closely in the fracture clinic by the orthopedic team to ensure healing of this rare type of fracture. (Figures 5 and 6 show the slow and delayed healing of the fracture six and 12 months, respectively, after the operation.)

## Discussion

Fragility or insufficiency fractures typically occur in the vertebrae, hip, distal radius, and the proximal humerus following minimal or no trauma but occur only rarely in the proximal femur. The subtrochanteric region of the femur is one of the strongest parts of the femur and is unlikely to fracture in low-energy trauma unless extreme osteoporosis is present.

Extensive studies have shown that therapy with bisphosphonates improves bone density and decreases fracture risk. However, increased bone density does not necessarily equate with good bone quality. Bone turnover is a natural part of maintaining bone health. By decreasing osteoclastic activity and bone resorption and therefore bone formation, micro-damage that occurs regularly in bone but that is normally repaired might accumulate after long-term use.

In a 2006 study that involved nine patients on long-term alendronate therapy, Odvenia *et al*. [3] found that severe suppression of bone turnover may occur, resulting in increased susceptibility to non-spinal fractures along with delayed healing. Although co-administration of estrogen or glucocorticoids appears to be a predisposing factor, this apparent complication can also occur with monotherapy. A conclusion drawn from this study was that bisphosphonates should be discontinued after five years and an alternative treatment should be considered [3,4]. This may explain why our patient had a side effect of bisphosphonates relatively early (after only four years of treatment) as she was on long-term corticosteroids, which can hasten the atypical fracture precipitation of risedronate.

Several case reports and case series have questioned the safety of long-term use of bisphosphonates. Most have described fractures that are (a) spontaneous, (b) subtrochanteric or midshaft femoral fracture, or (c) horizontal or oblique by less than 30 degrees or that have (d) cortical beaking and lateral or diffuse cortical thickening.

Those findings were found in patients who have been on long-term bisphosphonate treatment. The fractures also tend to show delayed union or non-union after orthopedic treatment. Our patient presented with a fracture that fits the above criteria. A limited number of reports state that alendronate-induced fractures occur after three to 10 years of regular use. Kwek *et al*. (2008) and Goh *et al*. (2007) [5,6] reported 17 women who were on long-term alendronate therapy (for a mean of five years) and who sustained low-energy subtrochanteric fractures [5-9].

It is important to stress that not everybody agrees on the safety of long-term use of bisphosphonates and some have even questioned the existence of bisphosphonate-precipitated atypical fractures. However, most of those studies were performed only to test the effectiveness of bisphosphonates within a relatively short period of time and did not involve a proper experimental randomized controlled trial to support or refute bisphosphonates as a cause of atypical fracture in the neck of the femur.

Cecilia *et al*. [10], for example, found, in a 2008 randomized control trial of 239 patients who had an average age of 81 and who had hip fracture, that one-year treatment with alendronate was effective in decreasing the bone resorption marker and increasing the bone density of the proximal femur. The authors also mentioned that the drug was well tolerated.

At a bone research meeting in 2008, researchers reported a large Danish registry cohort analysis showing that alendronate use was indeed significantly associated with an increased risk of atypical subtrochanteric fractures. The alendronate group had an incidence of subtrochanteric fractures of 2.9 out of 1000 patient-years, whereas controls had an incidence of only 1.6. However, regular hip fractures were also significantly more common among the alendronate group, suggesting that the alendronate-treated group had weaker bones than controls in the first place [11].

In a 2009 population-based registry that Abrahamsen *et al*. [12] performed to evaluate hip and subtrochanteric and diaphyseal femur fractures and compare their incidence in alendronate users with that in non-users, the atypical fracture type described above was found to be more common in alendronate users. Given the known efficacy of alendronate in reducing the rate of hip fracture, the higher rates of those atypical fractures among alendronate users were attributed to the increased use of alendronate among high-risk patients rather than an increase in risk associated with alendronate. The authors concluded that there was no evidence of an increased risk of fracture of the subtrochanteric or diaphyseal femur with either short- or long-term use of bisphosphonates [9,12].

In 2010, Black *et al*. [9] performed a secondary analysis using the results of three large randomized bisphosphonate trials: the Fracture Intervention Trial (FIT), the FIT Long-Term Extension (FLEX) trial, and the Health Outcomes and Reduced Incidence with Zoledronic Acid Once Yearly (HORIZON) Pivotal Fracture Trial. The authors reviewed 284 records for hip or femur fractures among 14,195 women in these trials and found that the estimated numbers of women who would need to be treated were 90 to prevent one hip fracture, 35 to prevent one non-vertebral fracture, and 14 to prevent one morphometric vertebral fracture. Thus, treating 1000 women with osteoporosis for three years would prevent about 100 fractures: 71 vertebral fractures and 29 non-vertebral fractures, including 11 hip fractures. On the basis of their studies, the estimated annual rate of subtrochanteric and diaphysial fractures attributed to bisphosphonate treatment is 2.3 per 10,000 patients. The authors concluded that the risk of subtrochanteric or diaphyseal femur fracture associated with bisphosphonate use is very low, even in women who both had osteoporosis and received bisphosphonates for up to 10 years [9].

With the increasing evidence of the characteristic atypical fracture of femur and the potential implications of long-term use of bisphosphonates, there has been a search for the best alternative drug to bisphosphonates when the latter are contraindicated. According to guidelines of the National Institute for Health and Clinical Excellence, strontium ranelate and raloxifene should be used as a second-line agent in the above situation. However, in atypical femur fracture attributed to bisphosphonates, Schneider [11] supported the use of teriparatide because of its osteoblaststimulating activity. Denosumab, which is a monoclonal antibody that reduces osteoclast activity, has been licensed in the United Kingdom, can be used for patients who are intolerant or not suitable for bisphosphonates, and is being used in our patient. It is worth mentioning that denosumab is still a new drug and the knowledge regarding its long-term safety is still relatively limited. However, the use of denosumab is associated with a small risk of osteonecrosis of the jaw, a similar complication of bisphosphonates, which Schneider and others [2,11,13] associate with atypical fracture of the femur.

## Conclusions

Bisphosphonates have been used widely and are still being used widely in the treatment of osteoporosis. However, use of these medications over a long period of time is possibly associated with atypical fractures. So it is important to set a time scale for the use of bisphosphonates to prevent such disastrous and rare side effects. We recommend a maximum treatment period of five years because the therapeutic value of bisphosphonates will last for 10 years. Other measures to treat and prevent osteoporosis – like a well-balanced diet, weight-bearing exercises, and calcium and vitamin D supplementation – should be continued and encouraged.

Large-scale randomized studies are needed in order to improve our understanding of the long-term safety of bisphosphonates and to reach a universal agreement for an alternative medication to manage patients with bisphosphonate-associated fractures, as these are very rare and may require more prolonged and complex treatment. These patients usually require additional evaluation and treatment along with surgical fixation. This might include bone scans to detect other stress fractures, stopping alendronate therapy, and referral to specialists in treating these unusual cases.

## Consent

Written informed consent was obtained from the patient for publication of this case report and accompanying images. A copy of the written consent is available for review by the Editor-in-Chief of this journal.

## Abbreviation

FIT, Fracture intervention trial.

## Competing interests

The authors declare that they have no competing interests.

## Authors’ contributions

AA revised and wrote the case report. ET wrote the case presentation and reviewed the case report. FR and JS looked after the patient and reviewed the case report. KN and PT reviewed the case report. All authors read and approved the final manuscript.

## References

[B1] National Institute for Health and Clinical Excellence Alendronate, etidronate, risedronate, raloxifene and strontium ranelate for the primary prevention of osteoporotic fragility fractures in postmenopausal women (amended) [www.nice.org.uk/guidance/TA160]

[B2] National Institute for Health and Clinical Excellence Alendronate, etidronate, risedronate, raloxifene, strontium ranelate and teriparatide for the secondary prevention of osteoporotic fragility fractures in postmenopausal women (amended) [www.nice.org.uk/guidance/TA161]

[B3] Odvina CV Zerwekh JE Rao DS Maalouf N Gottschalk FA Pak CY Severely suppressed bone turnover: a potential complication of alendronate therapy J Clin Endocrinol Metab 2005 90 1294 13014 15598694 10.1210/jc.2004-0952

[B4] Schneider JP Should bisphosphonates be continued indefinitely? An unusual fracture in a healthy woman on long-term alendronate Geriatrics 2006 61 31 33 16405362

[B5] Kwek EB Goh SK Koh JS Png MA Howe TS An emerging pattern of subtrochanteric stress fractures: a long-term complication of alendronate therapy? Injury 2008 39 224 231 10.1016/j.injury.2007.08.036 18222447

[B6] Goh SK Yang KY Koh JS Wong MK Chua SY Chua DT Howe TS Subtrochanteric insufficiency fractures in patients on alendronate therapy: a caution J Bone Joint Surg Br 2007 89 349 353 10.1302/0301-620X.89B3.18146 17356148

[B7] Ali T Jay RH Spontaneous femoral shaft fracture after long-term alendronate Age and Ageing 2009 38 625 626 10.1093/ageing/afp106 19556326

[B8] Sayed-Noor AS Sjödén GO Subtrochanteric displaced insufficiency fracture after longterm alendronate therapy-a case report Acta Orthopaedica 2008 79 565 567 10.1080/17453670710015580 18766492

[B9] Black DM Kelly MP Genant HK Palermo L Eastell R Bucci-Rechtweg C Cauley J Leung PC Boonen S Santora A de Papp A Bauer DC Fracture Intervention Trial Steering Committee HORIZON Pivotal Fracture Trial Steering Committee Bisphosphonates and fractures of the subtrochanteric or diaphyseal femur N Engl J Med 2010 362 1761 1771 10.1056/NEJMoa1001086 20335571

[B10] Cecilia D Jódar E Fernández C Resines C Hawkins F Effect of alendronate in elderly patients after low trauma hip repair Osteoporos Int 2009 20 903 910 10.1007/s00198-008-0767-z 18956132

[B11] Schneider JP Bisphosphonates and low-impact femoral fractures: current evidence on alendronate-fracture risk Geriatrics 2009 64 18 23 19256578

[B12] Abrahamsen B Eiken P Eastell R Subtrochanteric and diaphyseal femur fractures in patients treated with alendronate: a register-based national cohort study J Bone Miner Res 2009 24 1095 1102 10.1359/jbmr.081247 19113931

[B13] National Institute for Health and Clinical Excellence Denosumab for the prevention of osteoporotic fractures in postmenopausal women, NICE guidelines [www.nice.org.uk/guidance/TA204]

